# Use of Cerebrolysin in combination with high‐dose aspirin antithrombotic treatment of ischemic stroke: A case report

**DOI:** 10.1002/ccr3.4863

**Published:** 2021-09-24

**Authors:** Alexander Borisevich, Konstantin Veremeyuk

**Affiliations:** ^1^ Brest City Emergency Hospital Brest Belarus

**Keywords:** aspirin, cerebrolysin, ischemic stroke, posterior cerebral artery thrombosis

## Abstract

This clinical case is an example of a potential synergistic neuroprotective interaction of Cerebrolysin and high doses of aspirin. The case describes the significant recovery of neurological deficits in a patient with moderate ischemic stroke (NIHSS 12 points) caused by right posterior cerebral artery thrombosis (RPCAT) after treatment with Cerebrolysin in combination with high dose of aspirin. Within 7 days after the initiation of the treatment regimen, the NIHSS score improved to 3 points and a control MRI that was performed on the 10th day after stroke, showed a significant decrease of the ischemic area and cerebral edema zone. It further might have an evidence that the combination of Cerebrolysin with high‐dose aspirin is safe and might have a synergistic effect in the treatment of ischemic stroke.

## Graphical abstract

The combined treatment of ischemic stroke with the high‐dose aspirin and Cerebrolysin could act synergistically and contribute to a more complete neurological deficit recovery through the combined neuroprotective and neurotrophic effects

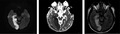

## 1 INTRODUCTION

Acute ischemic stroke is the second leading cause of death and one of the most common causes of adult disability worldwide.^1^ Thrombolytic therapy within 4.5 hours after stroke onset can significantly reduce mortality and morbidity. However, the effect is negligible after 4.5–6 hours and useful for only a small portion of patients.^1^ Therefore, many therapeutic strategies have been developed targeting the pathophysiological cascade that starts with ischemia and ultimately leads to irreversible tissue damage.^2^ One of the most effective neuroprotective drugs used in the acute phase of ischemic stroke is Cerebrolysin, which consists of low molecular weight peptides and free amino acids and has been shown to exert both neuroprotective and neurotrophic effects ^1^, ^2^, ^3^, ^4^. A meta‐analysis of these studies confirmed the beneficial effect of Cerebrolysin on the early recovery of neurological deficits in patients with acute ischemic stroke.^1^, ^5^, ^6^ Acetylsalicylic acid (aspirin) is widely used as secondary preventive drug in stroke therapy. In ischemic stroke, aspirin in high doses of 160–326 mg is used to achieve antithrombotic as well as neuroprotective effects.^7^ Antithrombotic treatment is an effective management tool for acute ischemic stroke and early secondary prevention as it reduces the risk of recurrent ischemic stroke with a low risk of hemorrhagic complications.^7^ The combination of high doses of aspirin and Cerebrolysin may have a potential effect in cases of ischemic stroke.

### 1.1 Patient Information

A 72‐year‐old male patient, left‐handed, was taken to the hospital by ambulance with complaints of weakness in the left extremities, left‐sided visual field loss, speech impairment, nausea, and dizziness which appeared immediately after awakening. The patient had a history of arterial hypertension (grade 2, risk 4) which was not properly controlled by antihypertensive medication. As the exact time of stroke onset was not known, the patient was not eligible for thrombolytic therapy.

### 1.2 Clinical Findings

Upon admission, vital signs showed an increase in blood pressure (180/110 mm Hg), while the heart rate (65 bpm) and the respiration rate (18 per minute) were within normal limits. The neurological examination revealed the following features: transcortical sensory aphasia, left‐sided homonymous hemianopia, mild left‐sided central facial nerve palsy, moderate dysarthria, left‐sided central hypoglossal nerve palsy, mild left‐sided hemiparesis (4/5), a pathological Babinski sign on the left, and left‐sided hemi‐lateral hemihypesthesia. The NIHSS score was 12 points. The mRS score was 2.

### 1.3 Diagnostic assessment

The laboratory and instrumental examinations were made after the patient's admission to the neurological unit in order to identify possible causes of the disease, as well as to exclude other pathologies with similar clinical features.

Blood tests revealed relative and absolute monocytosis, absolute basophilia, platelet size decrease, increased levels of total and conjugated bilirubin, an increase in prothrombin time as well as an increased fibrinogen level. In addition, an increase in total cholesterol level (6.7 mmol / L) was observed, which, according to the SCORE table for European regions indicates a very high risk of developing fatal cardiovascular diseases for this patient (≥10%).^8^

**FIGURE 1:**
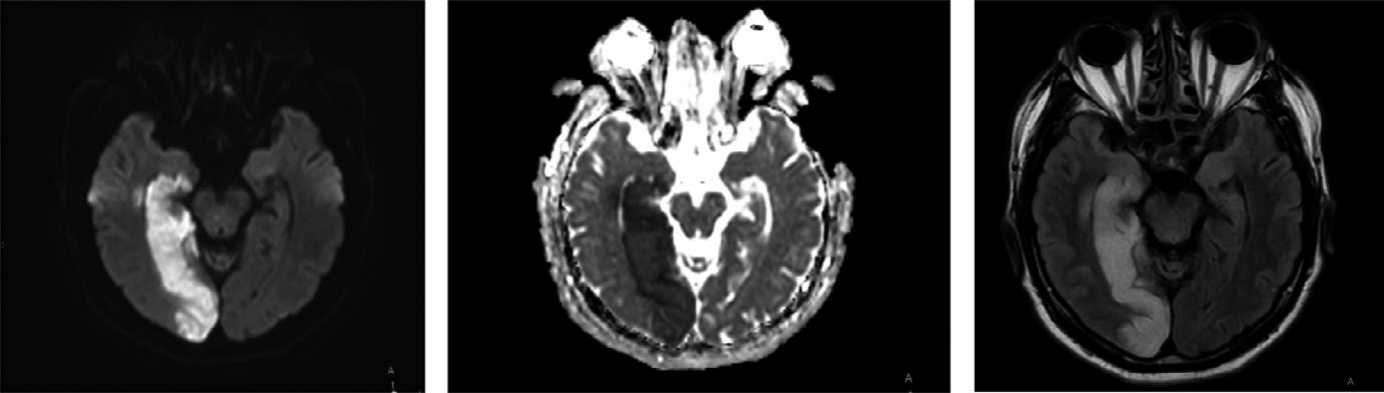
MRI images of the brain in axial projection with an edema and ischemia zone in the right posterior cerebral artery blood supply area

The patient underwent brain magnetic resonance imaging, on which the diffusion restriction zone without clear contours and smoothing of the adjacent furrows was found in the right temporo‐occipital region, measuring 30 × 90 × 40 mm (Figure 1). A focus with similar characteristics was also visualized in the right thalamus. MRI data confirmed the clinically diagnosed cerebral infarction in the RPCAT basin.

Subsequently, the patient underwent ultrasound examination of the brachiocephalic arteries, which revealed stenosis of the bifurcation of the right common carotid artery (30–40%) and the left common carotid artery (30%).

### 1.4 Therapeutic Intervention

After hospitalization of the patient in the stroke unit, antithrombotic and neuroprotective therapy was initiated: 30ml Cerebrolysin intravenously once daily for 16 days and oral administration of high‐dose aspirin (326 mg) once daily for 9 days, followed by low dose Aspirin (75 mg). In addition, the patient received basic stroke therapy in accordance with the AHA‐ASA recommendations and the clinical protocol of the Ministry of Health of the Republic of Belarus. For the concomitant hypertension, candesartan (8 mg once daily) and indapamide (2.5 mg once daily) were administered. Due to the fact that the patient has a very high risk of developing fatal cardiovascular diseases, atorvastatin (40 mg once daily) was prescribed to lower cholesterol levels. Furthermore, the patient received gastroprotection, with 20mg pantoprazole once daily during the entire course of treatment.

### 1.5 Follow‐up and outcome section

**FIGURE 2:**
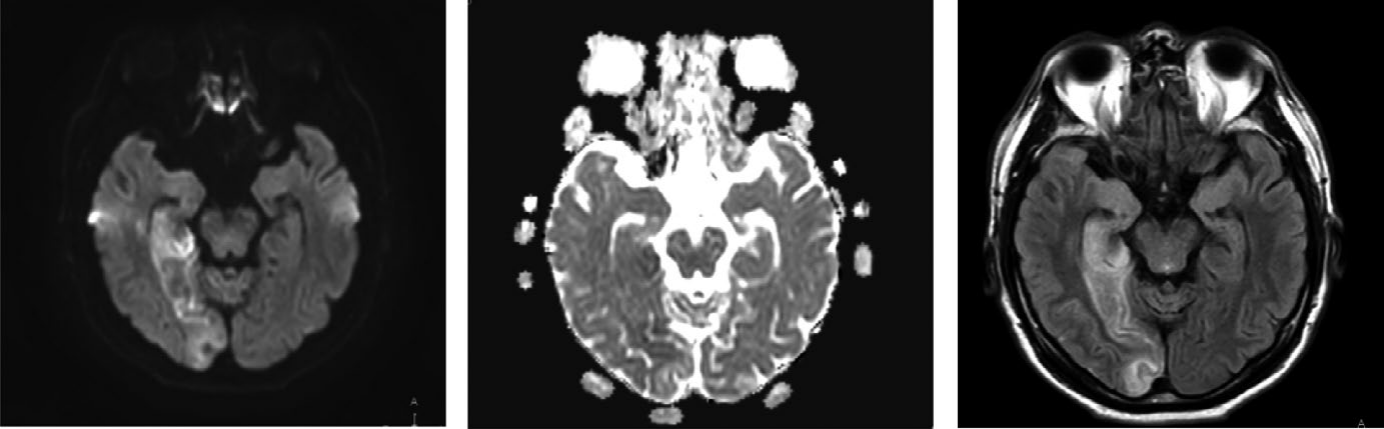
MRI images of the brain in axial view 10 days after stroke

Within 7 days after the initiation of the treatment regimen, the NIHSS score improved to 3 points, with remaining signs and symptoms of partial hemianopsia, hemiataxia, and mild central facial nerve palsy. The modified Rankin scale score (mRS) was 1 point. A control MRI was performed on the 10th day after stroke, which indicated a significant decrease of the ischemic area and cerebral edema zone (Figure 2).

At the time of the patient's discharge and after the last dose of Cerebrolysin on day 16 after stroke, the neurological status further improved and the residual deficits were limited to a partial left‐sided homonymous hemianopsia and a positive pathological Babinski reflex on the left. The NIHSS score further improved to 1 point, mRS score remained at 1 point.

## 2 DISCUSSION

This case is an example of a combination widely used: aspirin and the neuroprotective drug Cerebrolysin which can be applied in other similar cases. It further provides evidence that the combination of Cerebrolysin with high‐dose aspirin is safe and might have a synergistic effect in the treatment of ischemic stroke.

The clinical effectiveness of Cerebrolysin has repeatedly been demonstrated in randomized double‐blind placebo‐controlled multicenter studies.^1^, ^2^ A meta‐analysis confirms previous evidence that Cerebrolysin has a beneficial effect on early global neurological deficits in patients with acute ischemic stroke.^5^ These underlying mechanisms of Cerebrolysin are associated with the inhibition of calpain and consequently the stabilization of neuronal cells and with the induction of neuronal sprouting and neurogenesis. It has also been shown to affect a number of signaling pathways including the phosphatidylinositol 3‐kinase (PI3)/AKT pathway, the glycogen synthase kinase 3 beta (GSK3β) pathway, the Sonic hedgehog (Shh) pathway, and other pathways, which are responsible for the formation and maintenance of the blood‐brain barrier integrity.^4^

Antithrombotic treatment is an effective management tool for acute ischemic stroke and early secondary prevention as it reduces the risk of recurrent ischemic stroke with a low risk of hemorrhagic complications. This effect is mediated by inhibition of COX‐1 in the platelets preventing their adhesion to the vascular wall, as well as by the inhibition of acetylation of coagulation proteins which ultimately increases the rate of fibrinolysis.^7^ The neuroprotective effect of aspirin occurs once the blood flow in the thrombosed vessel is restored and is due to its inhibition of COX‐2 in brain tissue, a decrease of NOTCH‐1 gene expression as well as IL‐6 levels, which downregulates neuroinflammation.^9^, ^10^ Furthermore, aspirin has also been shown to interact with the ischemic cascade on the level of glutamate and to significantly decrease its levels post‐stroke.^9^

Therefore, the combined treatment of ischemic infarction with the simultaneous use of high‐dose aspirin and Cerebrolysin should act synergistically and contribute to a more complete neurological deficit recovery through the combined neuroprotective and neurotrophic effects of both agents. It further provides evidence that the combination of Cerebrolysin with high‐dose aspirin is safe and might have a synergistic effect in the treatment of ischemic stroke.

The use of Cerebrolysin in combination with high doses of aspirin may have been the main reason for the almost complete remission of neurological deficit in the patient with moderately severe stroke in the right posterior cerebral artery basin, which clearly justifies the further use of this combination in prospective studies and clinical practice.

## CONFLICT OF INTEREST

No conflict of interest to declare.

## AUTHOR CONTRIBUTIONS

KV involved in the treatment and clinical management decision‐making of the patient, obtained consent for the publication, and wrote draft manuscript. AB interpreted clinical data and critically revised the manuscript for important intellectual content.

## ETHICAL APPROVAL

Ethical approval was obtained from the director of Ambulance Hospital, Brest, Belarus. Formal approval is not necessary to report a case as "Case Report" in our hospital.

## CONSENT

Published with written consent of the patient.

## Data Availability

Data sharing is not applicable to this article as no datasets were generated or analyzed during the current study.
